# Association of human leukocyte antigen B genotypes with COVID-19 severity in Egyptian patients

**DOI:** 10.1038/s41598-026-36948-7

**Published:** 2026-02-17

**Authors:** Reham Abdelmonem, Heba Selim, Shymaa Abdullah Mohamed, Richard Donkor Amponsah, Mohamed Nabil Roshdy, Mona Hassan Hashish

**Affiliations:** 1 Microbiology Department, High Institute of Public Health, Alexandria University, Alexandria, Egypt; 2 Molecular Biology Unit, Applied Medical Chemistry, Medical Research Institute, Alexandria University, Alexandria, Egypt; 3 Department of Medical Microbiology, University of Ghana Medical School, Accra, Ghana; 4 Microbiology Department, General Military Hospital, Medical Military Academy, Alexandria, Egypt

**Keywords:** HLA-B alleles, Ct-value, COVID-19 severity, Egypt, Immunology, Microbiology, Molecular biology, Medical research

## Abstract

Host genetic factors, specifically major histocompatibility complex (MHC) influence the prognosis of COVID-19 infections. It plays a central role in the body’s ability to recognize and respond to pathogens, including viruses like SARS-CoV-2. This study aims to identify the significant human leukocyte antigen B (HLA-B) allele genotypes associated with severe cases among SARS-CoV-2-positive patients attending the PCR unit in The Fever Hospital-Alexandria, Egypt. This cross-sectional study was conducted from November 2022 through April 2023. Two swabs (nasopharyngeal and oropharyngeal) were collected from each patient for RT-PCR to determine the cycle threshold (Ct) value. Subsequently, 2 ml of EDTA blood was drawn from each patient for DNA extraction and HLA-B typing using the INNO-LiPA HLA-B Update Plus kit. Patients were divided according to the severity of their infection: mild (*N* = 15, mean Ct = 26.27 ± 1.27), moderate (*N* = 15, mean Ct = 24.20 ± 1.63), and severe (*N* = 15, mean Ct = 22.27 ± 0.93). Ct values were the lowest amongst the severe group with a mean of 22.27 (*p* = 0.026). Regarding the severity of SAR-CoV-2 infection, 39 HLA-B genotypes, 6 HLA-B super-types, and 22 HLA-B alleles were identified. HLA-B44 was the most significant allele when comparing the three groups of severity: mild, moderate, and severe *(p* = 0.044*)*. HLA-B08 and HLA-B44 had a statistical significance of *(p* = 0.011) and *(p* = 0.034*)* respectively when comparing the mild group to hospitalized group (severe + moderate). The Ct value is a clear predictor of SARS-CoV-2 severity. HLA-B44 and HLA-B08 were the most significant alleles associated with severe cases, emphasizing the association of human leukocyte antigen B alleles with disease severity and viral load in determining COVID-19 outcomes. These findings may contribute to the development of personalized medical management and improve strategies for COVID-19 control and prevention.

**Supplementary Information:**

The online version contains supplementary material available at 10.1038/s41598-026-36948-7.

## Introduction

The clinical presentation of SARS-CoV-2 infection varies widely, ranging from asymptomatic cases to severe disease that can lead to death. Many patients experience mild respiratory symptoms, while others develop serious complications requiring intensive care unit (ICU) admissions^1^. The severity of COVID-19 depends on a complex interplay of host, viral, and environmental factors, which collectively shape the immune response and disease outcome^2^. As a result, the World Health Organization (WHO) classified COVID-19 into three main categories: mild, moderate, and severe, based on the presence of clinical signs of viral pneumonia and hypoxia^3^. A key indicator of severe case is the cycle threshold (Ct) value, where low Ct values are linked to high viral loads^4^. Patients with high viral loads often experience worse symptoms and greater damage to extrapulmonary organs compared to those with higher Ct values and lower viral loads^5^. Moreover, elevated viral loads increase the risk of complications and mortality, making Ct value an important prognostic factor for predicting the course of COVID-19^6^.

The major histocompatibility complex (MHC), also known as human leukocyte antigen (HLA), is a genetic region located on chromosome 6 (6p21.3)^7^ that plays a vital role in the adaptive immune system. MHC molecules present antigens on cell surfaces to T cells, facilitating immune recognition and response^8^. The interaction between T cells and HLA alleles relies on the T-cell receptor’s ability to recognize specific configurations of the antigen-binding groove in HLA molecules^9^. MHC is divided into two classes: MHC class I (HLA-A, -B, -C) and MHC class II (HLA-DQ, HLA-DR and HLA-DP). HLA class I alleles, particularly HLA-B, are highly polymorphic, with thousands of variants at the A and B loci. These alleles are grouped into supertypes based on shared peptide-binding specificities^8,10^. The diversity of HLA alleles varies geographically and may influence infectious disease outcomes^11^, including COVID-19. Recent studies suggest that specific HLA alleles and supertypes may affect susceptibility to and prognosis of SARS-CoV-2 infection, alongside viral and environmental factors^12–14^. In Africa, the lower incidence of COVID-19 compared to other regions may be attributed to the diversity of HLA alleles present in the population^15^. As such, this study seeks to determine the significant HLA-B allele genotypes in confirmed SARS-COV-2 patients and also assess the relationship between viral load, HLA-B allele genotypes, and the severity of SARS-COV-2. Understanding these associations is crucial for developing personalized medical management plans and improving strategies for COVID-19 control and prevention.

## Methods

### Patients grouping

Patients were classified into mild, moderate, and severe groups according to the World Health Organization (WHO) COVID-19 clinical severity classification^16^. All patients were clinically evaluated by attending physicians at the Fever Hospital–Alexandria.

### Inclusion and exclusion criteria

Inclusion criteria: Adults aged ≥ 18 years with RT-PCR–confirmed SARS-CoV-2 infection who presented to the Fever Hospital–Alexandria during the study period and had complete clinical, laboratory, and radiological data required for severity classification.

Exclusion criteria: Patients with negative RT-PCR results, those younger than 18 years, individuals who had initiated COVID-19–specific treatment (antivirals, corticosteroids, or immunomodulatory drugs) before sample collection. Patients lacking complete medical records or those who declined consent were also excluded.

### Sample collection and processing

Nasopharyngeal and oropharyngeal swabs were collected at the time of presentation to the hospital, prior to the initiation of any COVID-19–specific treatment. All samples were taken within the first 24 h of clinical evaluation.Both swabs were immediately delivered to the hospital laboratory for RT- PCR. Peripheral venous blood (2 ml) was collected in EDTA tubes from each patient under a complete aseptic procedure. The blood samples were stored at −80 °C until the collection of all samples. None of the patients had received antiviral therapy, corticosteroids, or immunomodulatory drugs before sample collection.

### Extraction and amplification of viral RNA using real-time PCR

QIAamp Viral RNA mini kit (Qiagen, USA) and DiaPlexQ™ COVID-19 (N, ORF1a) detection Kit (SolGent, South Korea) were used for COVID-19 detection by quantitative real-time polymerase chain reaction (qRT-PCR) technique^17,18^. Extraction of viral RNA was done followed by amplification. The Ct values were determined by identifying the rising curve of these genes with their point of crossing the threshold^19^.

### Viral RNA extraction

For viral RNA extraction, 560 µl of prepared lysis buffer was mixed with 140 µl of saline from the nasopharyngeal swab. It was then vortexed for 15 s, followed by a 10-minute incubation at room temperature. Absolute ethanol (560 µl) was added, and the resulting mixture was vortexed. A portion of 630 µl of the mixture was applied to the QIAamp Mini spin column and centrifuged at 6000 x g for one minute. After adding 500 µl of Buffer AW1 and centrifuging, 500 µl of Buffer AW2 was added and centrifuged at 14,000 x g for three minutes. The column was dried by centrifuging in a new wash tube for one minute. Finally, 60 µl of elution buffer (AVE) was added, incubated for three minutes, and centrifuged at 10,000 x g. The purity of the eluted viral RNA was assessed using a Jenway™ NanoDrop, with an optimal OD 260/280 ratio between 1.8 and 2.1 indicating suitable purity.

### Amplification of viral RNA

The extracted viral RNA was directly used for amplification by qRT-PCR for the targeted gene of COVID-19 (N, ORF1a). COVID-19 (N, ORF1a) was detected according to the instructions inserted in the DiaPlexQ™ COVID-19 (N, ORF1a) Detection Kit (SolGent, South Korea). The PCR master mix for each sample was prepared by combining 10 µL of One Step qRT-PCR Buffer, 2 µL of One Step qRT-PCR Enzyme mix, and 3 µL of Primer & Probe Mixture (ORF1a) to a total of 15 µL. After vortexing and spinning down the mix, 5 µL of the template was added and sealed before spinning down again. The PCR tubes were then placed in a Rotorgene Q (Qiagen, USA) and subjected to a thermal profile consisting of reverse transcription at 50 °C for 15 min, initial PCR activation at 95 °C for 15 min, followed by 45 cycles of denaturation at 95 °C for 20 s and annealing/extension at 60 for 40 s. The qRT-PCR analysis targeted the SARS-CoV-2 N gene and ORF1a gene, with a cutoff Ct value of ≤ 40 indicating a positive result, while the internal control RNase P had a cutoff of < 40 for valid results and ≥ 40 for invalid results.

## Human leukocyte antigen (HLA) B typing

### Blood genomic DNA extraction

The genomic DNA was extracted from blood samples using a blood genomic DNA extraction Kit (spin column) (Solarbio, China)^20^. 750 µL of Red Blood Cell Lysis Buffer was added to 250 µL of blood, mixed, and incubated before centrifugation. The resulting pellet was treated with Solution A and Proteinase K, followed by Solution B, and processed through an adsorption column. The column was washed with a mix of washing buffer and absolute ethanol, air-dried, and eluted with 40 µL of preheated elution buffer. Finally, the purity of the eluted DNA was assessed using a Jenway™ NanoDrop, aiming for an OD 260/280 ratio between 1.8 and 2.1.

### Nucleic acid amplification

**Fig. 1:**
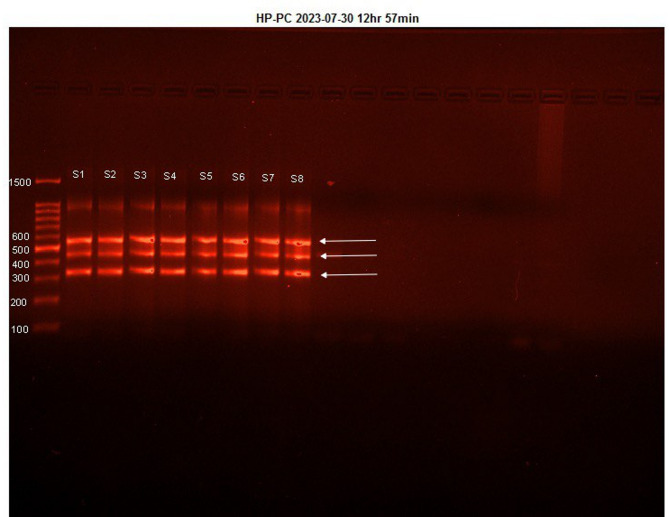
DNA gel electrophoresis of multiplex PCR amplification of exons 2 to 4 of *HLA-B* alleles. A 2% agarose gel stained with ethidium bromide was used to confirm amplification. Lanes S1–S8 contain amplified PCR products, with first lane showing a DNA molecular weight marker (Std). A negative control (distilled water without DNA) was included to confirm the absence of contamination (not visible in cropped image; see Supplementary Fig. 1 for full gel). Cropped sections are displayed for clarity; the original uncropped gel with visible edges is provided in Supplementary Fig. 1. No lanes were spliced or combined from different gels. Image brightness was uniformly adjusted for presentation.

The PCR reaction for each sample was prepared by mixing 12 µL of autoclaved distilled water, 5 µL of amplification buffer, 5 µL of HLA-B Multiplex primer solution, and 1.5 µL of LiPA-Taq, totaling 23.5 µL. To this master mix, 5 µL of genomic DNA preparation was added. Distilled water without DNA was used for the negative control. The samples were amplified using a pre-heated and calibrated thermal cycler. Finally, a 2% agarose gel was used to confirm the amplification of Exons via gel electrophoresis (Fig. 1).

### Gel electrophoresis and imaging

PCR products were resolved on a 2% agarose gel in 1× TAE buffer at 5 V/cm for 40 min, alongside a 100 bp DNA ladder (Thermo Fisher catalog number: 15628019, 100–1500 bp range). Gels were stained with ethidium bromide (0.5 µg/mL), visualized under UV light.

### Molecular typing of the HLA-B alleles

**Fig. 2:**
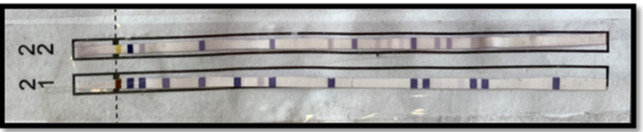
Strip (1) and Strip (2). Brown line on strip 1 and chrome line on strip.

Molecular typing was performed using the INNO-LiPA HLA-B Update Plus kit (Fujirebio Europe, Belgium)^20,21^. The procedure for denaturation and hybridization involved adding 10 µL of denaturation solution to each test trough, followed by 10 µL of the amplicon, and allowing denaturation at 25 °C for 5 min. A pre-warmed hybridization solution (2 mL at 56 °C) was added, and the INNO-LiPA HLA-B Update Plus strips were incubated at 56 °C for 30 min. Afterward, the strips were washed with 2 mL of pre-warmed stringent wash solution at 56 °C for 2 min and again for 10 min. For color development, each strip was washed twice with 2 mL of rinse working solution, incubated with 2 mL of conjugate working solution for 30 min, and then washed again before adding 2 mL of substrate working solution for 5–7 min. The strips were then washed in distilled water, dried, and read for results, with a positive indication marked by a clear purple/brown band (Fig. 2), assisted by the LiRAS software for interpretation.

### Statistical analysis

Statistical analysis was performed by SPSS V29.01.0. The normality of data distribution was assessed using the Shapiro-Wilk test. Continuous variables were expressed as mean ± standard error (SE). If the data followed a normal distribution (*p* > 0.05), parametric tests were used for comparison. If not (*p* < 0.05), non-parametric tests were applied. Categorical data were presented as frequencies and percentages. The Mann-Whitney test was used to compare continuous data between two groups, while the chi-square (x^2^) test was applied for categorical variables. A p-value < 0.05 was considered statistically significant.

.

## Results

### Demographics

The relationship between human leukocyte antigen B (HLA-B) allele genotypes, viral load, and disease severity in COVID-19 was assessed. The study included 45 patients with RT-PCR-confirmed SARS-CoV-2 infections. Patients were equally distributed into mild (*n* = 15), moderate (*n* = 15), and severe (*n* = 15) conditions based on the WHO clinical classification of COVID-19 severity. There were 29 females (64%) and 16 males (36%) in this cross-sectional study. The descriptive analysis of the biographical and clinical characteristics of SARS-CoV-2 infected patients showed no statistical significance between gender, clinical manifestations, clinical comorbidities, and COVID-19 severity (*p* > 0.05) (Table 1).

**Table 1:** Biographical and clinical characteristics of COVID-19 patients according to the severity of infection.

Parameter	Severity			*p*-value
	Mild (*n* = 15)	Moderate (*n* = 15)	Severe (*n* = 15)	
Gender				
Female	9 (60%)	11 (73.3%)	9 (60%)	> 0.05
Male	6 (40%)	4 (26.7%)	6 (40%)	
Clinical Manifestations				
Cough	10 (67%)	9 (60%)	8 (53%)	> 0.05
Dyspnoea	10 (67%)	9 (60%)	8 (53%)	
GIT (N, D) ^+^	6 (40%)	6 (40%)	3 (20%)	
Fever ≥ 38 °C	8 (53%)	15 (100%)	15 (100%)	
Fatigue	15 (100%)	15 (100%)	15 (100%)	
Comorbidities				
Asthma	1 (6.7%)	0 (0%)	2 (13.3%)	> 0.05
Chronic lung disease	0 (0%)	0 (0%)	1 (6.7%)	
Diabetes mellitus	1 (6.7%)	2 (13.3%)	0 (0%)	
Hypertension	0 (0%)	1 (6.7%)	2 (13.3%)	
No comorbidities	13 (86.7%)	12 (80%)	10 (66.7%)	

^+^ N; nausea, D: diarrhea.

* ***p*** ≤ **0.05** were considered significant at level.

### Normality testing

The Shapiro-Wilk test was conducted to assess the normality of the dataset. The test results indicated that ALT, AST, Ct values, and inflammatory markers (D-dimer, CRP) did not follow a normal distribution (*p* < 0.05). Therefore, non-parametric statistical tests were used for these variables. Conversely, age, lymphocyte count, platelet count, and electrolyte levels (Na, K) followed a normal distribution (*p* > 0.05), allowing for the use of parametric statistical tests.

### Radiological characteristics of COVID-19 patients

The radiological characteristics of COVID-19 patients were analyzed according to the severity of SARS-CoV-2 infection and summarized in Table 2. CT chest showed Ground-glass opacity (GGO) in 7 (47%) patients in the moderate group and 15 (100%) in the severe group. Patients in the mild group did not develop pneumonia with a statistically significant difference between severe and moderate groups (*p* = < 0.001).

**Table 2:** Radiological characteristics of COVID-19 patients according to the severity of infection.

CT chest (GGO)	Severity		
	Mild (*n* = 15)	Moderate (*n* = 15)	Severe (*n* = 15)
Yes	0 (0%)	7 (47%)	15 (100%)
No	15 (100%)	8 (53%)	0 (0%)

### Age and laboratory characteristics of COVID-19 patients

A significant association between the age of patients and the severity of infection when comparing the severe group to mild and moderate groups (*p* = 0.013) was found (Table 3). At the same time, Ct values showed that their mean levels were 26.27 ± 1.27, 24.20 ± 1.63, and 22.27 ± 0.93 in mild, moderate, and severe groups, respectively with a statistically significant difference between Ct values of the severe group compared to the mild group (*p* = 0.026). Also, there was a significant association between COVID-19 severity and both the lymphocyte count (*p =* 0.019), and platelets count (*p =* 0.007) when comparing severe to mild groups, in addition to the statistical significance of platelets count when comparing the moderate to mild severity groups (*p =* 0.036). Liver function tests showed a significant association between the severity of COVID-19 and elevation of both ALT and AST (*p <* 0.05). A significant correlation between the inflammatory markers D-dimer and CRP, and severity of infection when comparing both mild and moderate groups to severe group (*p <* 0.001) was found. The mean levels of electrolytes; Na and K, in the blood of COVID-19 patients, showed a statistically significant difference when comparing the severe group to the mild group (*p =* 0.016, *p =* 0.004), respectively.

**Table 3:** Age and laboratory tests of COVID-19 patients according to the severity of infection.

Parameters	Mild (mean ± S.E)	Moderate (mean ± S.E)	Severe (mean ± S.E)	*P* value
Age	29.53 **±** 1.70	31.33 **±** 2.61	52.67 **±** 6.60	0.917
				0.013^a^
				0.013^b^
CT value	26.27 **±** 1.27	24.20 **±** 1.63	22.27 **±** 0.93	0.22
				0.026^a^
				0.661
Lymphocytes	2113.47 **±** 261.35	2141.33 **±** 309.62	1432.53 **±** 210.91	0.803
				0.019^a^
				0.101
Platelet	291.07 **±** 20.82	240.00 **±** 18.58	211.33 **±** 22.67	0.036^a^
				0.007^a^
				0.11
Haemoglobin (mg/dl)	12.89 **±** 0.50	12.85 **±** 0.41	12.32 **±** 0.44	0.819
				0.533
				0.533
ALT (IU/L)	26.67 **±** 2.56	29.27 **±** 2.82	58.33 **±** 2.98	0.575
				<.001^a^
				<.001^b^
AST (IU/L)	24.20 **±** 2.14	29.33 **±** 2.07	62.20 **±** 2.82	0.074^a^
				<.001^a^
				<.001^b^
D-dimer (mg/dL)	0.57 **±** 0.12	0.52 **±** 0.08	2.01 **±** 0.16	0.901
				<.001^a^
				<.001^b^
CRP (mg/L)	16.31 **±** 4.63	10.87 **±** 1.33	53.31 **±** 6.30	0.901
				<.001^a^
				<.001^b^
Urea (mg/dL)	32.47 **±** 2.33	28.80 **±** 2.15	40.20 **±** 6.03	0.371
				0.442
				0.135
Creatinine (mg/dL)	0.73 **±** 0.04	0.75 **±** 0.04	1.06 **±** 0.14	0.849
				0.231
				0.295
Na (mmol/L)	141.33 **±** 0.98	140.00 **±** 0.87	137.47 **±** 1.04	0.38
				0.016^a^
				0.173
K (mmol/L)	4.47 **±** 0.14	4.21 **±** 0.15	3.85 **±** 0.09	0.197
				0.004^a^
				0.091

a statistically significant when compared to the mild group,.

b statistically significant when compared to the moderate group.

*p* ≤ 0.05 were considered significant at level.

Test of significance Manwatny U test between each two groups.

### HLA-B genotypes, supertypes, and alleles among the COVID-19 patients

#### Frequency of HLA-B genotypes and supertypes among COVID-19 patients

The frequency of HLA-B genotypes (*n* = 39) is shown in Table 4, whereas the frequency of HLA-B supertypes (*n* = 6) with HLA-B alleles (*n* = 22) is represented in Table 5.

**Table 4:** Frequency and distribution of HLA-B genotypes among severity groups.

HLA-B Genotype (*n* = 39)	Mild	Moderate	Severe
HLA-B* 14/15	1	0	0
HLA-B* 14/35	0	0	1
HLA-B* 14/40	0	0	1
HLA-B* 14/45	1	0	0
HLA-B* 15/35	0	1	0
HLA-B* 15/41	0	0	1
HLA-B* 15/42	1	0	0
HLA-B* 15/78	0	1	0
HLA-B* 18/41	0	0	1
HLA-B* 18/53	1	0	0
HLA-B* 27/35	1	0	0
HLA-B* 35/38	1	0	0
HLA-B* 35/41	0	1	0
HLA-B* 35/49	0	1	0
HLA-B* 35/53	0	0	1
HLA-B* 37/40	0	0	1
HLA-B* 38/44	0	2	1
HLA-B* 39/41	0	0	1
HLA-B* 41/49	0	0	1
HLA-B* 41/50	2	0	0
HLA-B* 41/52	1	0	0
HLA-B* 41/8	0	0	1
HLA-B* 42/52	2	0	0
HLA-B* 44/44	0	1	0
HLA-B* 44/49	1	0	0
HLA-B* 44/8	0	1	0
HLA-B* 49/57	1	1	0
HLA-B* 50/51	0	1	1
HLA-B* 50/52	1	0	0
HLA-B* 51/51	0	0	1
HLA-B* 7/14	0	1	0
HLA-B* 7/8	0	0	1
HLA-B* 8/15	0	1	0
HLA-B* 8/35	0	0	1
HLA-B* 8/40	1	0	0
HLA-B* 8/41	0	0	1
HLA-B* 8/44	0	1	0
HLA-B* 8/45	0	1	0
HLA-B* 8/49	0	1	0

**Table 5:** HLA-B supertype and allele distribution among severity groups.

Supertype association	HLA-B allele	Severity		
		Mild (*n* = 30)	Moderate (*n* = 30)	Severe (*n* = 30)
B07	HLA-B*7	0	1	1
	HLA-B*35	2	3	3
	HLA-B*42	3	0	0
	HLA-B*51	0	1	3
	HLA-B*53	1	0	1
	HLA-B*78	0	1	0
	HLA-B*15	2	3	1
B08	HLA-B*8	1	5	4
B27	HLA-B*14	2	1	2
	HLA-B*27	1	0	0
	HLA-B*38	1	2	1
	HLA-B*39	0	0	1
B44	HLA-B*18	1	0	1
	HLA-B*37	0	0	1
	HLA-B*40	1	0	2
	HLA-B*41	3	1	6
	HLA-B*44	1	6	1
	HLA-B*45	1	1	0
	HLA-B*49	2	3	1
	HLA-B*50	3	1	1
B58	HLA-B*57	1	1	0
B62	HLA-B*52	4	0	0

### Statistical analysis of HLA-B supertypes and alleles among COVID-19 patients

There was no statistically significant difference in the distribution of HLA-B supertypes associated with COVID-19 severity; mild, moderate, and severe (Table 6). The HLA-B supertypes in association with the mild group (*n* = 30) and the hospitalized group (merged moderate and severe (*n* = 60), showed that the supertypes; HLA-B07, HLA-B08, and HLA-B044 had statistical significance (*p* = 0.05), (*p* = 0.011), and (*p* = 0.033) respectively (Table 7). HLA-B044 was the most significant allele when comparing the three groups of severity; mild, moderate, and severe *(p* = 0.044*)* (Table 8). HLA-B08 and HLA-B044 were statistically significant (*p* = 0.011) and (*p* = 0.034), respectively when comparing the mild group to hospitalized group (merged moderate and severe groups) (Table 9).

**Table 6:** HLA-B supertype association with COVID-19 severity groups (mild, moderate, and severe).

Supertype association	Severity			*p*-value
	Mild (*n* = 30)	Moderate (*n* = 30)	Severe (*n* = 30)	
B07	8	9	9	0.962
B08	1	5	4	0.273
B27	4	3	4	0.913
B44	12	12	13	0.973
B58	1	1	0	1
B62	4	0	0	1

* *P-values* were considered significant at level ≤ 0.05.

**Table 7:** HLA-B supertypes association with the severity groups (mild and hospitalized groups).

Supertype association	Severity		*p*-value
	Mild (*n* = 30)	Moderate + Severe (*n* = 60)	
B07	8	18	0.05*
B08	1	9	0.011*
B27	4	7	0.366
B44	12	25	0.033*
B58	1	1	1
B62	4	0	1

* P ≤ 0.05 were considered significant at level.

**Table 8:** Statistical analysis of HLA-B allele frequency among COVID-19 patients (mild, moderate, and severe).

HLA-B allele (*n* = 22)	Severity			*p*-value
	Mild (*n* = 30)	Moderate (*n* = 30)	Severe (*n* = 30)	
HLA-B*7	0	1	1	1
HLA-B*8	1	5	4	0.273
HLA-B*14	2	1	2	0.819
HLA-B*15	2	3	1	0.607
HLA-B*18	1	0	1	1
HLA-B*27	1	0	0	1
HLA-B*35	2	3	3	0.882
HLA-B*37	0	0	1	1
HLA-B*38	1	2	1	0.779
HLA-B*39	0	0	1	1
HLA-B*40	1	0	2	0.564
HLA-B*41	3	1	6	0.15
HLA-B*42	3	0	0	1
HLA-B*44	1	6	1	0.044*
HLA-B*45	1	1	0	1
HLA-B*49	2	3	1	0.607
HLA-B*50	3	1	1	0.449
HLA-B*51	0	1	3	0.317
HLA-B*52	4	0	0	1
HLA-B*53	1	0	1	1
HLA-B*57	1	1	0	1
HLA-B*78	0	1	0	1

*P ≤ 0.05 were considered significant at level.

**Table 9:** Statistical analysis of HLA-B alleles among COVID-19 patients; mild, and hospitalized groups.

HLA-B allele	Severity		*P*-value
	Mild (*n* = 30)	Hospitalized group (*n* = 60)	
HLA-B*7	0	2	1
HLA-B*8	1	9	0.011*
HLA-B*14	2	3	0.655
HLA-B*15	2	4	0.414
HLA-B*18	1	1	1
HLA-B*27	1	0	1
HLA-B*35	2	6	0.157
HLA-B*37	0	1	1
HLA-B*38	1	3	0.317
HLA-B*39	0	1	1
HLA-B*40	1	2	0.564
HLA-B*41	3	7	0.206
HLA-B*42	3	0	1
HLA-B*44	1	7	0.034*
HLA-B*45	1	1	1
HLA-B*49	2	4	0.414
HLA-B*50	3	2	0.655
HLA-B*51	0	4	1
HLA-B*52	4	0	1
HLA-B*53	1	1	1
HLA-B*57	1	1	1
HLA-B*78	0	1	1

* *P* ≤ 0.05 were considered significant at level.

## Discussion

Recent research has focused on the correlation between specific HLA alleles and how severe COVID-19 can be, with several studies indicating that certain alleles may either increase susceptibility or provide protection against SARS-CoV-2. As such, understanding these associations can be crucial for developing personalized medical management plans and possibly improving COVID-19 control and prevention, especially in Africa where such studies have been relatively scarce. Our study identified the significant human leukocyte antigen B (HLA-B) allele genotypes associated with severe cases among SARS-CoV-2-positive patients attending the PCR unit in The Fever Hospital-Alexandria, Egypt. We enrolled 45 patients with RT-PCR-confirmed SARS-CoV-2 infection and among these, 29 females were divided into three groups: mild (9), moderate (11), and severe (9) groups. The remaining 16 males were also categorized into mild (6), moderate (4), and severe (6) groups. Our statistical analysis showed no significant association between gender and severity of COVID-19 (*p* > 0.05) likely because the men and women in our sample had similar age distribution and comorbidity patterns. Other factors such as sample size and the mix of mild and moderate cases may also reduce the ability to detect differences between sexes. Abdelhafiz et al.^22^. and Raimondi et al.^23^. found no statistical significance between gender and COVID-19 in Egypt and Italy respectively. In contrast to these results, a meta-analysis study suggested that males were more likely to develop severe COVID-19 infection than females^24^.

Moderate and severe cases were combined for specific statistical comparisons to increase sample size and improve the reliability of associations with HLA-B alleles. While this combination may reduce statistical power in some cases, it was justified based on the lack of significant differences in key variables between these two groups (*p-values* for ALT, AST, and Ct values showed a progressive but non-significant difference between moderate and severe cases). Additionally, previous studies have combined similar severity groups for genetic association analyses when shared pathophysiological mechanisms were observed. However, for individual comparisons where differences were significant, we maintained separate groups to ensure accuracy and avoid potential bias.

Comorbidities such as asthma, chronic lung disease, diabetes mellitus, and hypertension were evaluated in relation to SARS-CoV-2 and they showed no statistical significance (*p* > 0.05). This contrasts with the study by Albadawy et al.^25^., where multiple comorbidities were associated with severe clinical symptoms. This variance may be due to the differently assessed comorbidities in addition to the sample size and time frame of the current study. Ground-glass opacity (GGO) was identified in 73% of all chest CT scans. Specifically, GGO was present in 7 (47%) patients in the moderate group and all 15 (100%) patients in the severe group. Cozzi et al.^26^. highlighted that while GGO is a non-specific sign, it is the most frequently observed feature in COVID-19 cases. Moreover, GGO is not the only finding on chest CT scans as other features, such as lung consolidation, can also be observed. On the other hand, Frija-Masson et al.^27^. reported that 61.4% (60 non-severe and 29 severely ill patients) exhibited GGO, with no statistical significance. Similarly, Wu et al.^28^. found that all patients in severe group showed GGO, but again, this was not statistically significant.

The mean age was recorded at 29.53 ± 1.70 years for mild group, 31.33 ± 2.61 years for moderate group, and 52.67 ± 6.60 years for severe group, respectively with a statistical significance (*p* = 0.013) indicating that the magnitude of the infection increases with older age groups. This finding is in association with the results of Fraghaly et al.^29^. and Statsenko et al.^30^., where age was considered as a significant risk factor for critical COVID-19 cases. Furthermore, a study conducted by Jones et al.^31^. reported that children are not at risk of critical clinical cases of SARS-CoV-2 compared to adults. In contrast to these, Dudley et al.^32^. found that younger age groups were at a higher risk of severe COVID-19 cases due to lower compliance with social distancing compared to adults. The strong association between older age and severity in our study is consistent with most evidence because aging reduces immune function and increases chronic conditions. Studies that do not find this link usually have younger cohort or limited numbers of severe cases.

The Ct value is inversely correlated with SARS-CoV-2 viral load, hence the lower the Ct value, the higher the viral load. A high Ct value suggests a high number of nucleic acid replication cycles are required for the fluorescent signal to cross the threshold to detect the low viral load. In our study, the mean Ct value was at 26.27 ± 1.27 in the mild group, 24.20 ± 1.63 in the moderate group, and 22.27 ± 0.93 in the severe group. This means that the viral load in the severe group was higher than the mild and moderate groups with a statistical significance (*p* = 0.026). Comparably, a study concluded on the use of Ct values as a clinical tool to predict COVID-19 severity (*p* < 0.0001)^33^. Although lower Ct-values were significantly associated with increased severity in our study, other studies^34,35^ have found no correlation between Ct-values and disease severity. Therefore, Ct-value should be interpreted with caution and not considered a definitive predictor of severity in all populations.

We also found correlation between lymphopenia and the magnitude of SARS-CoV-2 infection, with the mean value of lymphocyte count being lower in the severe group (1432.53 ± 210.91) compared to the mild group (2113.47 ± 261.35). Both Toori et al.^36^. and Wang et al.^37^. found that peripheral lymphocyte counts were a good predictor of COVID-19 severity. Our statistical analysis showed a significant association between platelet count and the severity of infection between mild to severe groups (*p =* 0.007) and mild to moderate groups (*p =* 0.036). Similar studies have reported a link between lower platelet count and an increased risk of developing severe COVID-19^38,39^. In Egypt, a study by El-Khaiat et al.^40^. suggests that platelet count, as an easily accessible parameter, should be considered an indicator of COVID-19 severity due to the statistically significant difference in thrombocytopenia among the studied severity groups.

Liver injury in this study was evaluated by measuring ALT and AST levels. The mean ALT and AST levels were at the lowest in the mild group, measuring 26.67 ± 2.56 IU/L and 24.20 ± 2.14 IU/L, respectively. In the moderate group, ALT increased to 29.27 ± 2.82 IU/L, while AST rose to 29.33 ± 2.07 IU/L. The highest levels were observed in the severe group, with ALT at 58.33 ± 2.98 IU/L and AST at 62.20 ± 2.82 IU/L.T. There was an association between these enzyme levels and the magnitude of COVID-19 cases (*p* < 0.001). The rising levels of ALT and AST from mild to severe groups indicate the impact of liver injury on liver function tests (LFTs) in severe COVID-19 cases. Similar studies report elevated LFTs are significantly associated with moderate or severe disease^41^. Also, Shokri Afra et al.^42^. suggested that LFTs are potential prognostic biomarkers for patients in early SARS-CoV-2 infection. The mean levels of the inflammatory markers D-dimer and CRP were 0.57 ± 0.12 mg/dL and 16.31 ± 4.63 mg/L in the mild group, 0.52 ± 0.08 mg/dL and 10.87 ± 1.33 mg/L in the moderate group, and 2.01 ± 0.16 mg/dL and 53.31 ± 6.30 mg/L in the severe group, respectively, with a significant correlation between the magnitude of COVID-19 infection and the increased mean levels of both inflammatory markers. This is similar to a meta-analysis suggesting that inflammatory markers might be used to assess the severity and prognosis of SARS-CoV-2 infection^43^.

Although serum Na and K electrolyte mean levels were in the normal range, they showed an association when making a comparison between mild and severe groups. This is in relation with a study by Mohamed et al.^44^., where the mean serum sodium, and potassium, were within normal levels in SARS-CoV-2 infected patients. However, they were significantly higher in those with severe clinical presentation. Lippi et al.^45^. on the other hand proved that COVID-19 severity is associated with lower serum concentrations of Na, and K and recommended the measurement of electrolytes at initial presentation and serial monitoring during hospital admission to help with prognosis.

HLA-B supertypes B07, B08, and B44 were associated with the severity of COVID-19 when comparing the mild group to the hospitalized (moderate and severe) group. Guerini et al.^46^ and Alnaqbi et al.^47,48^ similarly reported associations between these supertypes and critical COVID-19 cases. On the contrary, although Luo et al.^49^. in their study found B44 supertype to be more frequent among SARS-CoV-2-infected patients, there was no significance correlation suggesting it may not be a strong determinant of critical cases in their cohort due to the potential variability in B44 role across populations. Individual allele analysis identified HLA-B44 to be significantly associated with diseases severity across all groups (*p* = 0.044), and both HLA-B8 (*p* = 0.011) and HLA-B44 (*p* = 0.034) when comparing mild to hospitalized groups. These findings are consistent with Migliorini et al.^50^ and Pisanti et al.^51^ who linked HLA-B08 and HLA-B44 to increased susceptibility to SARS-CoV-2 infection, potentially due to impaired presentation of viral epitopes, as suggested by Correale et al.^12^. In China, Zhang et al.^52^ reported an increased fold change in HLA-B44:03 in SARS-CoV-2-infected lung epithelial cells, further supporting its role in disease severity. These alleles may contribute to severity because they present a narrower or less effective set of SARS-CoV-2 peptides to CD8 T cells. Some populations do not show the same associations due to different allele frequencies and linkage with other immune genes. Our findings likely reflect population-specific immunogenetic patterns in the Egyptian context.

### Limitations

This study has some limitations.The relatively small sample size reduces the statistical power and limits the generalizability of our findings. The study was also conducted in a single center, which may not fully reflect the genetic diversity of the broader Egyptian population. In addition, we did not perform univariate or multivariate regression analyses. The analyses were limited to pairwise statistical comparisons between severity groups, which restricts our ability to adjust for potential confounding factors such as age, comorbidities, lymphocyte count, platelet count, and inflammatory markers. Furthermore, a healthy control group was not included because the study aimed to investigate HLA-B allele associations with COVID-19 disease severity among confirmed SARS-CoV-2–positive individuals. The clinical setting and ethical constraints during the pandemic period limited the ability to recruit healthy participants. Future multicenter studies with larger sample sizes and multivariate analysis with the inclusion of non-infected controls are needed to validate and expand upon these findings.

## Conclusion

In our cohort, lower Ct-values were associated with greater disease severity. HLA-B*44 and HLA-B*08 were the most significant alleles associated with severe cases, emphasizing the association of human leukocyte antigen B alleles with disease severity and viral load in determining COVID-19 outcomes. These findings may contribute to the development of personalized medical management plans and improve strategies for COVID-19 control and prevention.

## Supplementary Information

Below is the link to the electronic supplementary material.

Supplementary Material 1

## Data Availability

The data used during the current study are available from the corresponding author on reasonable request.

## Abbreviations

HLA-B: Human leukocyte antigen-B
MHC: Major histocompatibility complex
SARS-CoV-2: Severe acute respiratory syndrome coronavirus 2
COVID-19: Coronavirus disease 2019
Ct: Cycle threshold
RT-PCR: Reverse transcription polymerase chain reaction
CRP: C-reactive protein; ALT: Alanine aminotransferase
AST: Aspartate aminotransferase
GGO: Ground-glass opacity
RNA: Ribonucleic acid
DNA: Deoxyribonucleic acid
Na: Sodium
K: Potassium
LFTs: Liver function test
OD: Optical density
SPSS: Statistical package for the social sciences
