# Supplementary figures and images for: Association of human leukocyte antigen B genotypes with COVID-19 severity in Egyptian patients
Source: Sci Rep. 2026 Feb 17;16:7193. doi: 10.1038/s41598-026-36948-7 (PMC12921020; doi:10.1038/s41598-026-36948-7)

HP-PC 2023-08-15 11hr 05min

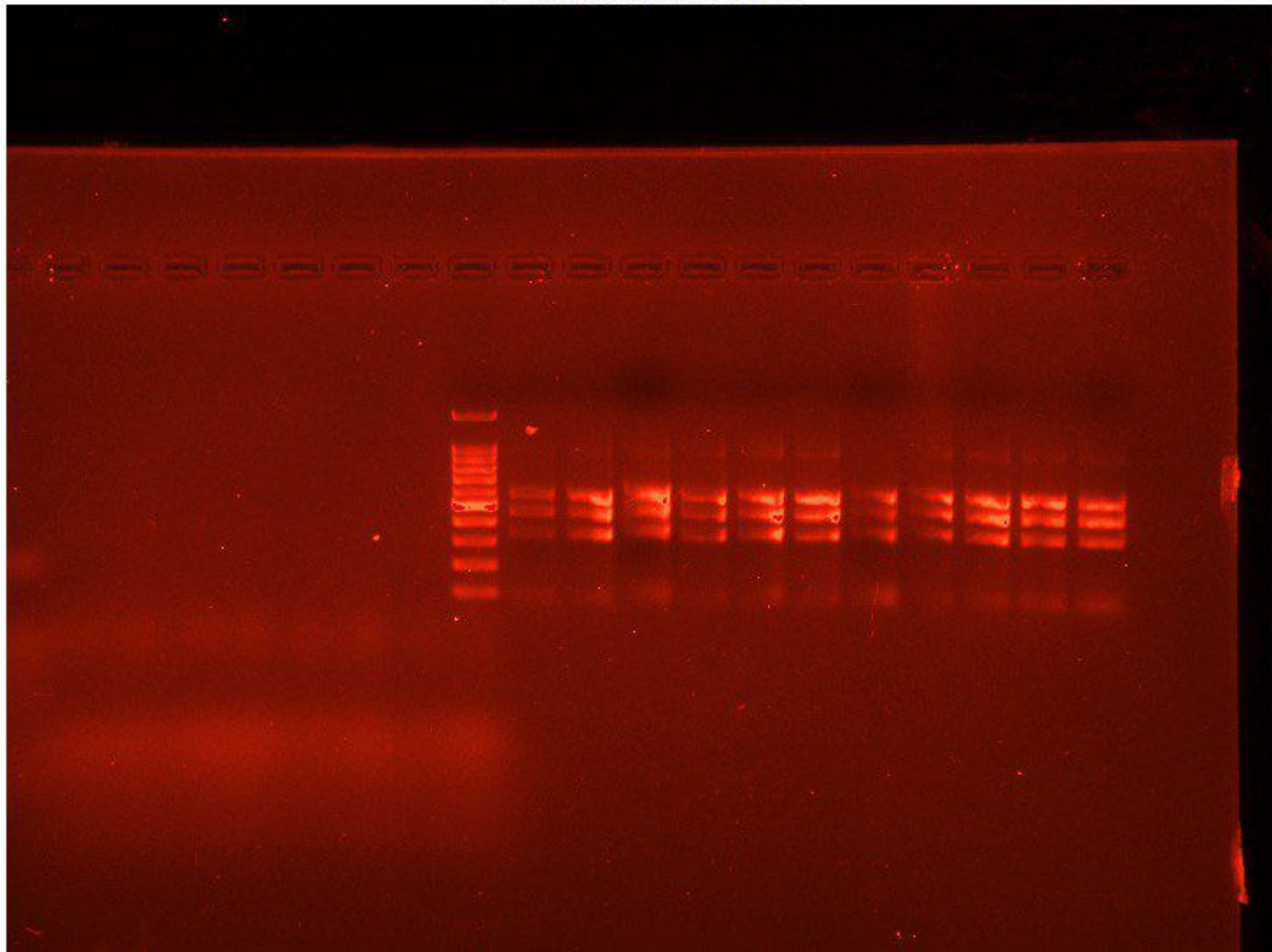

Supplement: Supplementary file 1 — Supplementary Material 1 [file 41598_2026_36948_MOESM1_ESM.pdf]
